# Time-varying association between blood pressure and malignant brain edema after large hemispheric infarction: a prospective cohort study

**DOI:** 10.1007/s10072-025-08147-1

**Published:** 2025-03-29

**Authors:** Xindi Song, Yanan Wang, Wen Guo, Meng Liu, Yilun Deng, Ming Liu

**Affiliations:** 1 Department of Neurology, West China Hospital, Sichuan University, 37 Guo Xue Xiang, Chengdu, 610041 China; 2 Center for Cerebrovascular Diseases, West China Hospital, Sichuan University, Chengdu, China; 3 Institute of Brain Science and Diseases, West China Hospital, Sichuan University, Chengdu, China; 4 Center of Gerontology and Geriatrics, West China Hospital, Sichuan University, Chengdu, 610041 China; 5 Department of Pediatrics, Baylor College of Medicine, Houston, TX USA

**Keywords:** Stroke, Cohort, Large hemispheric infarction, Edema, Blood pressure, Dynamic

## Abstract

**Aim:**

To investigate the time-varying association between blood pressure (BP) and malignant brain edema (MBE) risk after large hemispheric infarction (LHI).

**Methods:**

We prospectively enrolled LHI patients (CT hypodensity > 1/3 middle cerebral artery territory within 6 h or > 1/2 within 6-48 h of onset) from registry cohort. BP was recorded hourly and analyzed for 24 h and in two 12-hour epochs after onset: 1–12 h and 13–24 h. MBE was defined as neurological deterioration symptoms with midline shift ≥ 5 mm. Generalized estimating equation (GEE) compared BP patterns by MBE occurrence. Logistic regression and restricted cubic splines (RCS) assessed dose-response associations.

**Results:**

Among 414 included LHI patients (mean age 69 ± 14y, median onset-to-admission interval 3 h [interquartile range, IQR 2–5]), 117(28.3%) developed MBE with a median post-onset interval of 29 h (IQR 15–56). No significant difference in BP level was observed during 1–12 h. Systolic BP (SBP) was significantly higher in MBE group over time during 13–24 h (GEE *P* = 0.027). The 24-hour mean SBP exhibited a U-shaped association with MBE risk (nonlinear test, *P* = 0.029), with no significance in 1–12 h and a positive correlation between mean SBP in 13–24 h and MBE risk (aOR 1.02[1.00-1.04]). A threshold effect for 24-hour mean diastolic BP (DBP) was identified (non-linear test, *P* = 0.039), with DBP increase above 70 mmHg associated with higher MBE risk (aOR 1.03 [1.00–1.07]). Similarly, elevated mean DBP > 75mmHg during 1-12 h was associated with higher MBE risk.

**Conclusion:**

In LHI patients, 24-hour mean SBP demonstrated a U-shaped association with MBE risk, with no significant effect during 1–12 h and a positive linear correlation during 13–24 h after onset. DBP exhibited a threshold effect, primarily influencing MBE risk during 1–12 h after onset.

**Supplementary Information:**

The online version contains supplementary material available at 10.1007/s10072-025-08147-1.

## Introduction

Large hemispheric infarction (LHI) is among the most devastating forms of ischemic stroke, carrying the worst prognosis, with approximately one-third of cases progressing to malignant brain edema (MBE) [1, 2]. MBE is a life-threatening condition that can lead to brain herniation and death, with mortality rates reaching as high as 80% [3, 4]. While decompressive craniectomy could reduce mortality, more than half of survivors are left with severe disability [4, 5]. Given these challenges, preventing MBE at an early stage is more critical than relying solely on reactive treatments [6].

Cerebral autoregulation is disrupted after stroke onset, making blood flow in ischemic regions highly dependent on systemic blood pressure (BP) [7]. While previous studies reported associations between elevated admission systolic blood pressure (SBP) and brain edema development [8, 9], these investigations primarily focused on general stroke populations and did not specifically examine MBE. Moreover, previous studies measured BP at admission, failing to account for its temporal variations after stroke, which may influence outcomes differently over time [10].

Optimal BP management strategies for LHI patients remain undefined, particularly in relation to MBE prevention. We aims to bridge these gaps by investigating the time-varying association between BP and MBE risk in LHI patients, providing evidence to refine BP management and improve patient outcomes.

## Methods

### Study population

This study was nested within the stroke registry cohort of Department of neurology, West China Hospital, with ascertainment details available elsewhere [11]. We included LHI patients from January 1, 2016, to December 31, 2020. LHI was defined as an acute ischemic stroke with computed tomography (CT) scan showing a low-density area involving > 1/3 of middle cerebral artery (MCA) territory within 6 h of onset or > 1/2 of MCA territory within 6 to 48 h [12]. Detailed inclusion and exclusion criteria are provided in Online Resource 1.

### Clinical information

All patients underwent baseline evaluations to collect clinical information. Inportant time metrics were recorded, including intervals from onset to admission, LHI diagnosis, and MBE occurrence. We assessed three-month functional outcome by modifed Rankin Scale (mRS) to evaluate long-term prognosis. Details of clinical information were provided in Online Resource 2. Intra- and inter-rater agreement for imaging data was calculated was good (detailed in Online Resource 3,4).

### Blood pressure measurement

Systolic and diastolic blood pressure (DBP) were measured hourly from admission to 24 h after onset using the non-paralyzed limb. Baseline BP was defined as the initial measurement taken upon arrival, prior to imaging or treatment. For patients who developed MBE within 24 h, only BP measurements preceding MBE occurrence were kept, in order to ensure the appropriate sequential order of studied BP parameters and MBE. For each patient, mean, maximum, minimum, and range (difference between maximum and minimum) of systolic and diastolic blood pressure (DBP) were calculated for three epochs: 24 h, 1–12 h, and 13–24 h after onset.

### Evaluation of malignant brain edema

MBE was defined as space-occupying brain edema with midline shift of septum pellucidum ≥ 5 mm at fornix level on follow-up imaging within 7 days of onset or upon neurological deterioration, accompanied by worsening consciousness or unilateral pupil dilation; or need for decompressive craniectomy. MBE were independently evaluated by two trained neurologists (X.S and M.L). The intra- and inter-rater agreement of MBE evaluation was good (Online Resource 3,4).

### Statistical analysis

We summarized continuous variables as mean ± standard deviation (SD) or median (interquartile range [IQR]), and categorical variables as frequencies and percentages. Student’s t-test or Mann-Whitney U test was used for continuous variables, and Chi-square or Fisher’s exact test was applied for categorical variables, depending on sample size. To assess the distribution of three-month mRS score between groups, we additionally performed ordinal shift analysis. We applied generalized estimating equations (GEE) to compare BP trends over 24 h between MBE and non-MBE groups. We performed multivariable logistic regression to examine the association between BP and MBE, selecting confounders based on univariable analysis (*P* < 0.1), clinical relevance, and prior research [13]. We predefined subgroups by thrombectomy status and reperfusion success and performed logistic regression among subgroups. We used restricted cubic splines (RCS) analysis [14] to evaluate the dose-response relationship between BP and MBE. All tests were two-sided, with *P* ≤ 0.05 considered statistically significant. Full statistical methods are provided in Online Resource 5.

## Results

### Characteristics of participants

We finally included 414 eligible LHI patients (Online Resource 6). The mean age was 69 ± 14 years, with 50.0% (207/414) male. The median interval from onset to admission and LHI diagnosis was 3.12 h (IQR 2–5) and 12.92 h (IQR 4.82–31.32), respectively. Patients with MBE were more severe, more likely to receive thrombectomy and dehydration therapy, and more of them experienced in-hospital complications (All *P* < 0.05). MBE occurred in 28.26% (117/414) of patients, with median onset-to-occurrence of 28.97 h (IQR 15.42–56.10); 18.8% (22/117) developed MBE within 24 h. Three-month mRS was significantly higher in MBE patients (6 [IQR 4–6] vs. 4 [IQR 2–5], *P* < 0.001), and ordinal shift analysis further demonstrated that MBE patients had significantly higher odds of worse three-month functional outcome (OR 4.59, 95% CI: 3.00–7.02, *P* < 0.001). Online Resource 7 shows the detailed information of patients with and without MBE.

### Blood pressure characteristics over 24 h after LHI

We recorded 6016 SBP/DBP pairs, with an average of 15 ± 5 pairs per patient during 24 h, 7 ± 3 pairs during 1–12 h and 8 ± 3 pairs during 13–24 h after onset. At least three pairs were recorded in both 1–12 h and 13–24 h epochs for 90% (357/414) of patients, and at least six pairs over 24 h for 95% (385/414). Missing data were primarily due to delayed admission, interventional procedures, and imaging.

BP rose initially and then gradually declined after LHI. Around 13th hour, the decline slowed, and BP remained relatively stable thereafter with minor fluctuations (Online Resource 8). Baseline SBP and DBP were 141 mmHg (IQR 125–159) and 83 mmHg (IQR 72–95), with mean SBP and DBP during 24 h at 131.24 mmHg (IQR 121.86-143.64) and 75.64 mmHg (IQR 69.70-82.29), respectively.

### Association between SBP parameters and MBE in LHI patients

**Fig. 1:**
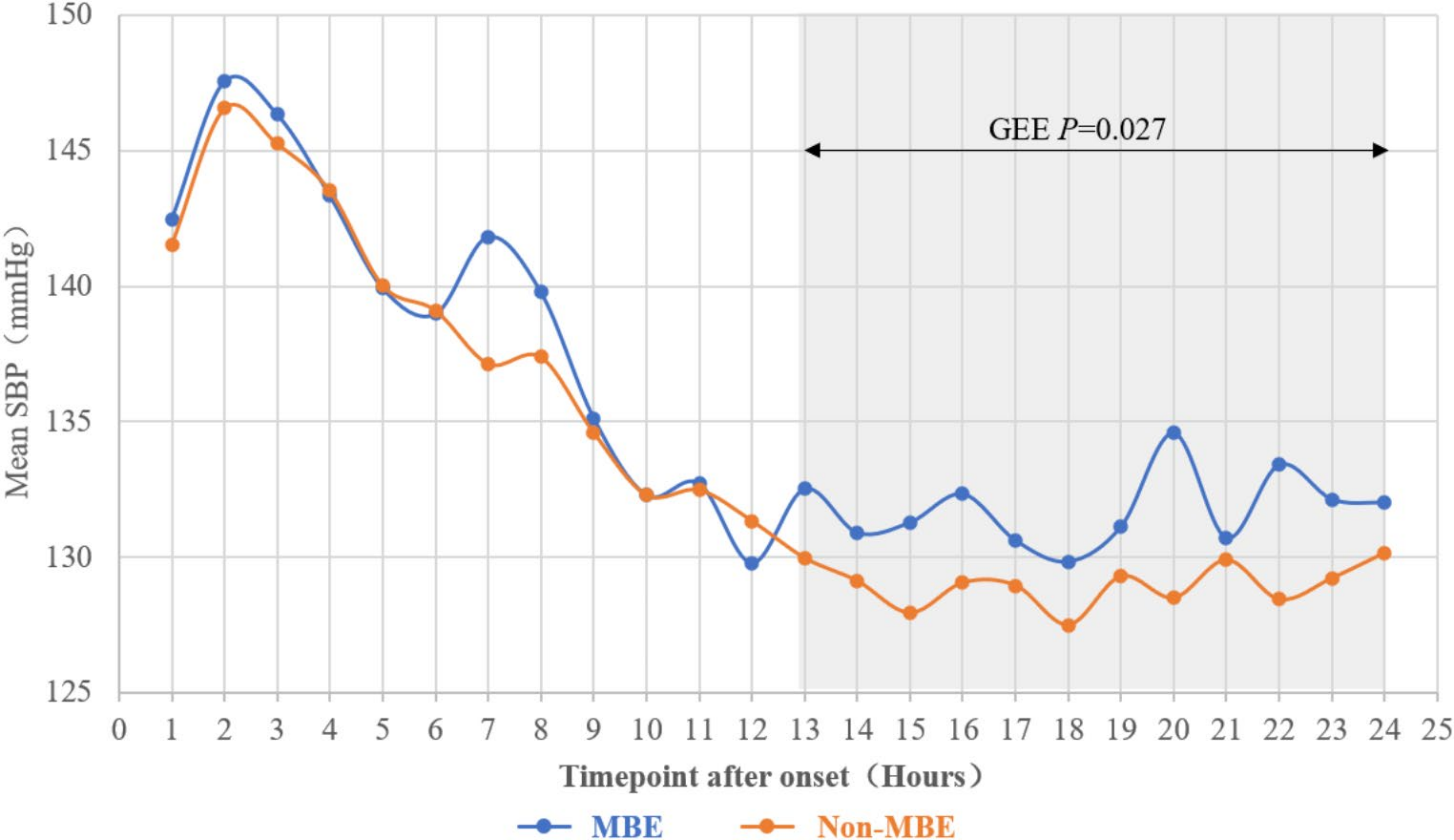
Overall SBP Pattern during 24 h after Large Hemispheric Infarction SBP, systolic blood pressure; MBE, malignant brain edema; GEE, generalized estimating equation

Figure 1 shows the 24-hour SBP pattern by MBE occurrence. GEE revealed no significant difference in SBP level between MBE and non-MBE groups during the first 12 h *(P* > 0.05), while SBP was significantly higher in MBE group during 13–24 h (*P* = 0.027).

During 24 h after onset, more patients with MBE had mean SBP ≥ 140 mmHg (41.03% vs. 29.29%, *P* = 0.022). During 13–24 h epoch, the proportion of patients with mean SBP ≥ 160 mmHg (11.11% vs. 4.04%, *P* = 0.007) and ≥ 180 mmHg (5.98% vs. 0.67%, *P* = 0.003) was significantly higher in MBE group. Detailed comparison of SBP parameters between patient with and without MBE is in Online Resource 9.

Multivariate logistic regression showed that higher 24-hour mean SBP (OR 1.02, 95% CI 1.00-1.04, *P* = 0.031), higher 24-hour minimum SBP (OR 1.02, 95% CI 1.00-1.03, *P* = 0.022), higher 13–24 h mean SBP (OR 1.02, 95% CI 1.00-1.04, *P* = 0.013), higher 13–24 h maximum SBP (OR 1.02, 95% CI 1.01–1.03, *P* = 0.009), and higher 13–24 h minimum SBP (OR 1.01, 95% CI 1.00-1.03, *P* = 0.044) were independently associated with MBE development (Online Resource 10). Consistent results were identified across subgroups (Online Resource 11).

**Fig. 2:**
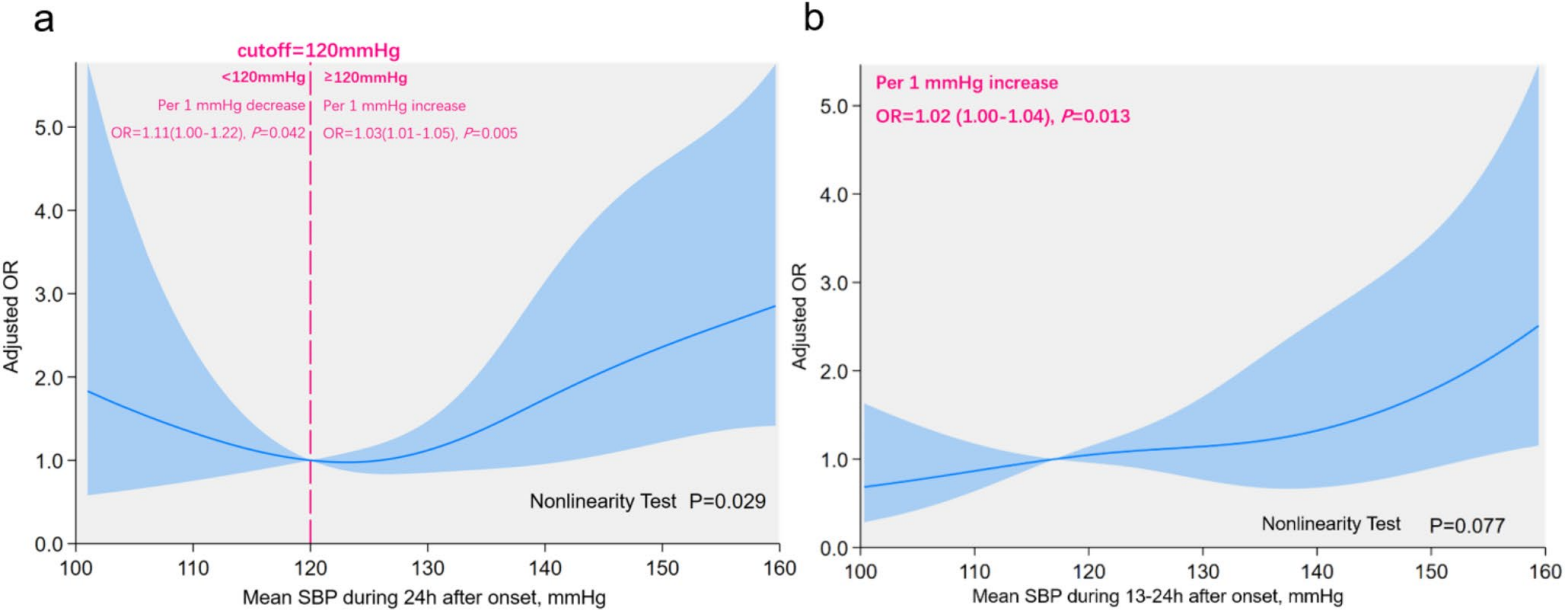
Association between SBP and MBE Risk in patients with large hemispheric infarction during different epochs after onset. The panels showed association between mean SBP during **a**) 24 h; **b**) 13–24 h after onset and MBE risk. The vertical axis represented adjusted OR, adjusted by age, sex, onset-to-admission interval, infarct size, NIHSS score, thrombectomy, and dehydration therapy. Solid blue line indicated OR values, while light blue shaded area represented the 95% confidence interval. The reference point was 120 mmHg at x-axis, with spline knots at the 5th, 35th, 65th, and 95th percentiles. SBP, systolic blood pressure; OR, odds ratio; NIHSS, National Institutes of Health Stroke Scale

RCS showed a U-shaped relationship between 24-hour mean SBP and MBE risk (non-linear test, *P* = 0.029, Fig. 2a). MBE risk declined as SBP decreased from higher levels, reaching a nadir at 123.4 mmHg, beyond which further reductions were associated with increased risk. Using 120 mmHg as a clinical threshold, each 1 mmHg increment above 120mmHg raised MBE odds by 3% (OR 1.03, 95% CI 1.01–1.05; *P* = 0.005), whereas each 1 mmHg decrement below 120 mmHg increased odds by 11% (OR 1.11, 95% CI 1.00-1.22; *P* = 0.042). No significant association was found between 1 and 12 h mean SBP and MBE (Online Resource 12). However, 13–24 h mean SBP demonstrated a linear positive dose-response with MBE risk (non-linear test *P* = 0.077, Fig. 2b).

### Association between DBP parameters and MBE in LHI patients

GEE analysis revealed no significant difference in overall DBP pattern between the MBE and non-MBE groups during 24 h after onset (*P* > 0.05, Online Resource 13). Compared to non-MBE group, the MBE group had a higher proportion of patients with 13–24 h mean DBP ≥ 100 mmHg (9.4% vs. 2.69%, *P* = 0.003) and ≥ 110 mmHg (5.98% vs. 1.35%, *P* = 0.008), as shown in Online Resource 14. Multivariate logistic regression identified that increased 24-hour mean DBP (OR 1.02, 95% CI 1.00-1.05, *P* = 0.049) and 1–12 h mean DBP (OR 1.02, 95% CI 1.00-1.04, *P* = 0.039) were independently associated with MBE development (Online Resource 15). Consistent results were only identified among non-thrombectomy and non-reperfusion subgroups (Online Resource 16).

**Fig. 3:**
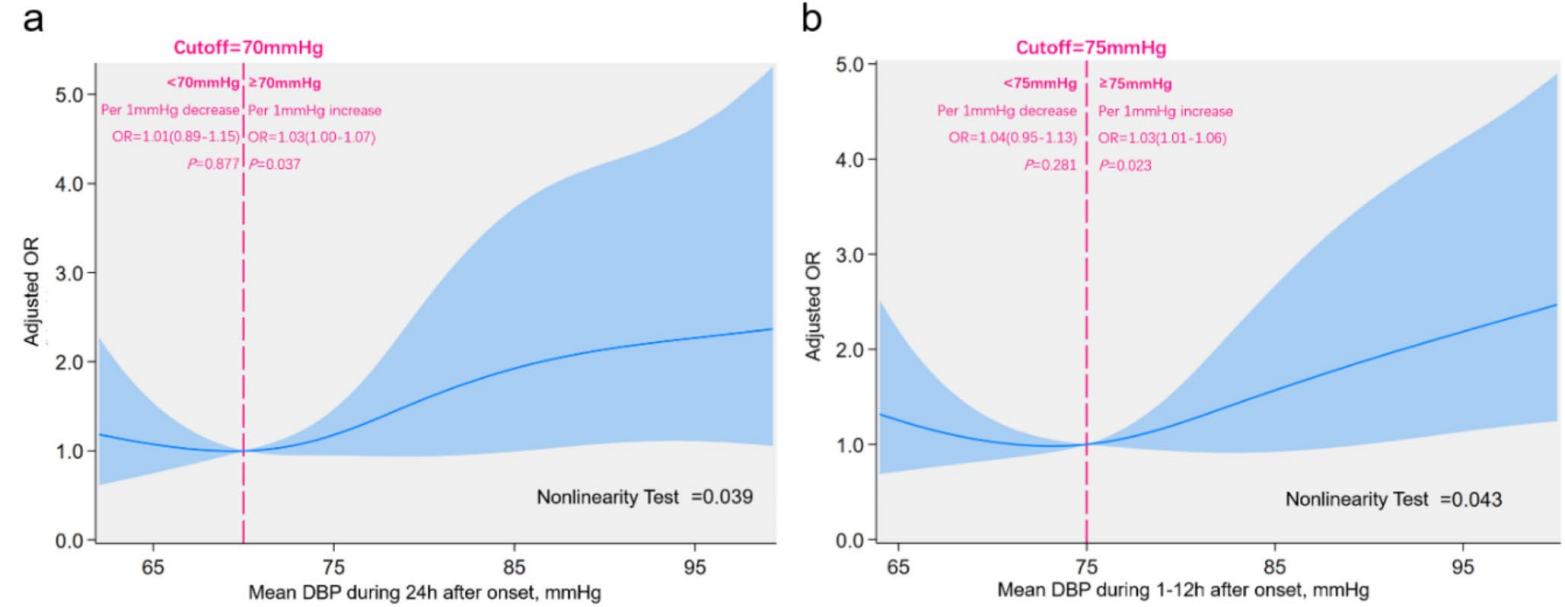
Association between DBP and MBE Risk in Patients with Large Hemispheric Infarction during Different Epochs after Onset. The panels showed association between mean DBP during **a**) 24 h; **b**) 1–12 h after onset and MBE risk. The vertical axis represented adjusted OR, adjusted by age, sex, onset-to-admission interval, infarct size, NIHSS score, thrombectomy, and dehydration therapy. Solid blue line indicated OR values, while light blue shaded area represented the 95% confidence interval. The reference point was 70 mmHg (**a**) and 75mmHg (**b**) at x-axis, with spline knots at the 5th, 35th, 65th, and 95th percentiles. DBP, diastolic blood pressure; OR, odds ratio; NIHSS, National Institutes of Health Stroke Scale

RCS showed a threshold relationship between 24-hour DBP and MBE risk (non-linear test, *P* = 0.039, Fig. 3a). As DBP declined from higher levels, MBE risk decreased, reaching a nadir at 69.4 mmHg, beyond which further changes had no significant effect. Employing 70 mmHg as a clinical threshold, each 1 mmHg increment above 70mmHg raised MBE odds by 3% (OR 1.03, 95% CI 1.00-1.07; *P* = 0.032), while decreases below 70 mmHg were linked to a non-significant increase in MBE risk (OR 1.01, 95% CI 0.89–1.15; *P* = 0.882). A similar threshold relationship was observed for 1–12 h mean DBP and MBE risk (non-linear test, *P* = 0.043, Fig. 3b), with the lowest risk at 73.2 mmHg and 75 mmHg as the cutoff. For 1–12 h mean DBP ≥ 75 mmHg, each 1 mmHg increase corresponded to a 3% rise in MBE odds (OR 1.03, 95% CI 1.01–1.06; *P* = 0.013). No significant association was detected between 13 and 24 h mean DBP and MBE risk (Online Resource 17).

## Discussion

In this study, we investigated the time-varying association between BP and MBE risk after LHI. We found that the influence of BP on MBE risk varied by time epochs: In LHI patients, 24-hour mean SBP demonstrated a U-shaped association, with no significant effect during 1–12 h but a positive linear correlation in 13–24 h after onset. DBP exhibited a threshold effect, primarily influencing MBE development during first 12 h post-onset. To our knowledge, this is the first study specifically addressing BP management in LHI and its association with MBE. These findings underscored the importance of considering temporal BP dynamics in optimizing MBE prevention strategies for LHI patients.

In our study, SBP declined in non-MBE group but remained elevated in MBE group during 13–24 h after onset, with a positive association identified between mean SBP and MBE risk during this period. No significant association was observed for SBP during 1–12 h epoch. This pattern aligned with previous studies. Shin et al. [10] and VISTA collaboration [15] reported a linear relationship between SBP and poor clinical outcomes during the 8-24-hour window. Prasad et al. [16] found that neurological deterioration was associated with greater SBP variability within first three days after thrombectomy, primarily during 12–24 h. This effect may result from progressive impairment of cerebral autoregulation, where elevated SBP exacerbates brain edema and intracranial pressure, increasing MBE risk. Alternatively, the progression of brain tissue edema and raised intracranial pressure during the late acute phase could trigger the Cushing reflex, further elevating SBP. In contrast, no significant association was observed between SBP during 1–12 h after onset and MBE, consistent with Shin et al. [10] and the VISTA collaboration [15], who reported an insignificant relationship between SBP and functional outcome or early neurological deterioration within first 8 h post-stroke. Cerebral autoregulation may remain partially intact during the initial post-stroke hours, potentially mitigating the impact of SBP on MBE risk [15, 17]. Our findings emphasized the critical importance of SBP management especially during the 13–24 h phase after onset to reduce MBE risk, while also highlighting the complexity of SBP’s role during acute phase and the need for further research to clarify its underlying mechanisms.

A U-shaped association was found between mean SBP and MBE risk during 24-hour period, with lowest risk at 120 mmHg. Previous studies linked higher admission SBP to MBE risk [9] and elevated 24-hour mean SBP to radiological evidence of brain edema [18]. However, they did not specifically focus on LHI, nor use the full MBE definition, which includes both clinical and radiological criteria. Our findings suggested that the optimal mean SBP range for LHI within 24 h after onset was around 120 mmHg, consistent with prior trials. Post-hoc analysis demonstrated that SBP targets below 140 mmHg were associated with better outcome compared to those below 180 mmHg [19], while more aggressive reductions below 120 mmHg after thrombectomy were linked to worse outcomes [20]. Elevated BP could disrupt the blood-brain barrier, causing brain edema [21], while excessively low BP could reduce cerebral blood flow, increasing ischemic injury [22].

We identified a threshold effect between DBP and MBE risk. Changes in 24-hour mean DBP below 70 mmHg were not linked to MBE development, while elevation in DBP above this threshold significantly raised MBE risk. A similar threshold of 75 mmHg was observed during 1–12 h epoch. The role of DBP in cardiovascular or cerebrovascular outcomes remains controversial, with some studies reporting a J-shaped relationship and optimal DBP ranges of 70–80 mmHg [23–26], while others showed a linear association [27, 28]. Research on DBP and MBE, particularly in LHI patients, was limited. We are the first to demonstrate this threshold effect, suggesting a broader safe range for DBP management compared to SBP. The time-dependent association likely reflected the importance of maintaining brain perfusion during the first post-onset 12 h, where DBP playing a greater role than SBP, as indicated by the formula: mean arterial pressure = 1/3 × (SBP + 2 × DBP). Notably, the association between DBP and MBE was significant only in patients without thrombectomy or successful reperfusion. Consistent with our findings, Hong et al. [29] reported that higher baseline BP correlated with better functional outcomes in patients with successful reperfusion but poorer outcomes in those without. Patients without thrombectomy treatment or successful reperfusion often presented later, had larger infarct areas, or experienced more severe symptoms, necessitating more precise blood pressure management. In such cases, maintain brain perfusion becomes more essential, with DBP playing a pivotal role.

In our study, the median interval from symptom onset to MBE occurrence was 28.97 h, consistent with prior research [30, 31]. Among the 117 MBE cases, 81.2% (95/117) occurred > 24 h after onset, and all BP measurements of these patients were included for analysis. For the remaining 22 patients developing MBE within 24 h, only pre-MBE BP data were kept to ensure correct temporal sequence between studied parameters and outcome, which ensured that the BP data analyzed were obtained strictly before MBE, making it more probable that BP variations influenced MBE development rather than the reverse. Nevertheless, our findings still indicate association rather than causation. Progressive cerebral edema, even before meeting MBE criteria, might influence BP through mechanisms like Cushing reflex [32, 33], suggesting a potential bidirectional or cyclical relationship. Further studies, including animal experiments and randomized controlled trials (RCTs), are needed to clarify causality.

Our study had several strengths. We are the first to focus on BP management in among LHI population. Currently, no specific recommendations for BP management in LHI are available. Our findings could serve as a guide for personalized BP management in this critically ill population and provide preliminary data for future RCTs. Second, we offered a detailed analysis of temporal BP dynamics and their link to MBE, taking time-varying effect of BP into account. Third, we identified key BP thresholds relevant to MBE after LHI, providing insights for targeted management. Lastly, we identified that while SBP affected MBE consistently among subgroups, DBP had a greater impact on non-reperfusion patients, highlighting the need for personalized management based on treatment and reperfusion status.

Our study had some limitations. First, not all patients had BP measured for 24 times, which was inevitable in studies analyzing dynamic indicators at multiple time points. However, 90% (357/414) had at least three measurements in both 1–12 h and 13–24 h epochs, and 95% (385/414) had at least six measurements during 24 h. Previous studies on hemodynamic parameters within 12–72 h post-stroke included patients with 1–10 measurements, which was comparable to our study [6, 16, 34]. Second, pre-stroke BP, which influences post-stroke BP elevation [35], was unavailable due to patients’ limited habit of regular monitoring. However, we substituted baseline BP and hypertension history, which partially reflect pre-stroke BP level. Third, we excluded patients developing space-occupying hemorrhagic transformation before MBE occurrence, which may introduce selection bias. However, other reasons of mass effect interfere with MBE assessment, and previous studies also excluded such patients [13, 36]. Fourth, some potentially influential factors, such as laboratory parameters, were not available and should be included in future studies. Fifth, as an observational study, our findings demonstrated associations rather than causation. Although we excluded post-MBE BP data and most MBE cases occurred > 24 h after BP measurement, reverse causality (e.g., via Cushing reflex) cannot be fully excluded. Further RCTs and animal studies are needed to clarify causality.

## Conclusion

In LHI patients, 24-hour mean SBP demonstrated a U-shaped association with MBE risk, with no significant effect during 1–12 h and a positive linear correlation during 13–24 h after onset. DBP exhibited a threshold effect, primarily influencing MBE risk during 1–12 h after onset.

## Electronic supplementary material

Below is the link to the electronic supplementary material.

Supplementary Material 1

## Data Availability

The data that support the findings of this study are available from the corresponding author upon reasonable request.
