# Supplementary material for: Time-varying association between blood pressure and malignant brain edema after large hemispheric infarction: a prospective cohort study
Source: Neurol Sci. 2025 Mar 29;46(8):3727–34. doi: 10.1007/s10072-025-08147-1 (PMC12267317; doi:10.1007/s10072-025-08147-1)
Supplement: Supplementary file 1 — Supplementary Material 1 [file 10072_2025_8147_MOESM1_ESM.docx]

##### Supplementary Methods

**Time-varying Association between Blood Pressure and Malignant Brain Edema after Large Hemispheric Infarction: A Prospective Cohort Study**

Xindi Song^1,2,3^ , Yanan Wang^1,2,3^, Wen Guo^1,4^, Meng Liu^1,2,3^, Yilun Deng^1,5^, and Ming Liu^1,2,3^

Correspondence to Ming Liu, MD, PhD, Department of Neurology, West China Hospital, Sichuan University, 37 Guo Xue Xiang, Chengdu, China 610041, email liuming201502@163.com

Journal: Neurological Sciences

**Online Resource 1. Study Population**

Inclusion criteria included: (1) age ≥18 years; (2) presented within 24h of onset; (3) pre-stroke modified Rankin Scale (mRS) score 0–1.

Exclusion criteria included: (1) space-occupying hemorrhagic transformation or other causes of mass effect observed on imaging within 7 days; (2) lack of follow-up head imaging within 7 days; (3) bilateral infarctions; (4) anterior or posterior cerebral artery involvement; (5) absence of BP measurements within 24h of onset or before MBE occurrence.

**Online Resource 2. Clinical Information**

1. **Demographic information:** Age, sex
2. **Time metrics:** Interval from stroke onset to admission, large hemispheric infarction (LHI) diagnosis, and malignant brain edema(MBE) occurrence
3. **Vascular risk factors:**

**Hypertension**: A previous confirmed diagnosis of hypertension, a documented history of antihypertensive medication use, or three separate measurements of resting blood pressure (on different days) showing a systolic blood pressure (SBP) >140 mm Hg and/or a diastolic blood pressure (DBP) >90 mm Hg[1]。

**Diabetes**: A previous confirmed diagnosis of diabetes, a documented history of antidiabetic medication use, a fasting blood glucose level ≥7.0 mmol/L, or a random blood glucose level or a 2-hour post-oral glucose tolerance test (OGTT) blood glucose level ≥11.1 mmol/L[2]。

**Hyperlipidemia**: A previous confirmed diagnosis of hyperlipidemia, a documented history of lipid-lowering medication use, a total cholesterol level ≥6.22 mmol/L, or a triglyceride level ≥2.27 mmol/L[3]。

**Atrial Fibrillation**: A documented diagnosis of atrial fibrillation, or electrocardiogram evidence of chronic or paroxysmal atrial fibrillation during this hospitalization[4]。

**Coronary Artery Disease**: Myocardial ischemia, hypoxia, or infarction caused by stenosis or occlusion of the coronary arteries as confirmed by coronary angiography prior to the current onset[5]。

1. **Stroke Severity:** National Institutes of Health Stroke Scale (NIHSS) score[6], infarct size at LHI diagnosis (classified by the proportion of low-density area in the middle cerebral artery[MCA] territory on computerized tomography[CT], 1/3-1/2, 1/2-2/3, or >2/3 MCA territory) and the Alberta Stroke Program Early CT Score (ASPECTS)[7, 8]. Infart size and ASPECTS were independently evaluated by two trained neurologists (X.S and Q.W), with good intra- and inter-observer agreement was good (detailed in eAppendix 2, Supplementary Table 1 below).
2. **Etiology:** TOAST (the Trial of Org 10172 in Acute Stroke Treatment) criteria classification[9] of ischemic stroke
3. **In-hospital Treatment:** Intravenous thrombolysis, mechanical thrombectomy, and dehydration therapy,
4. **In-hospital Complications:** Pneumonia (characterized by symptoms such as cough and sputum production, with abnormal lung auscultation findings like rales, crackles, or bronchial breath sounds, abnormal white blood cell count, and positive chest imaging findings) and urinary tract infection (characterized by urinary irritation symptoms such as suprapubic tenderness and dysuria, with or without fever, and a positive urine culture from a clean-catch midstream sample or non-catheterized specimen).
5. **Functional outcome:** Three-month functional outcome was modified by the modified Rankin Scale (mRS).

**Online Resource 3. Assessment of Inter- and Intra-Rater Agreement in Imaging Evaluations**

Two neurologists (X.S. and M.L.) independently reviewed brain images from 20 patients in a blinded, sequential manner to calculate inter-rater agreement. Intra-rater agreement was assessed by comparing X.S.’s evaluations at two separate time points using the same 20 patients. The agreement assessment included admission Alberta Stroke Program Early CT Score (ASPECTS), infarct size at diagnosis of large hemispheric infarction (LHI) (categorized as 1/3–1/2, 1/2–2/3, or >2/3 of middle cerebal artery[MCA] territory based on low-density area on computerized tomography[CT]), and existence of malignant brain edema (MBE) on imaging. We calculated intraclass correlation coefficient (ICC) for agreement of ASPECTS, and Kappa for infarct size (weighted Kappa) and existence of MBE (unweighted Kappa). Agreement was classified as good (>0.80), substantial (0.61–0.80), moderate (0.41–0.60), or fair (<0.40) for kappa statistics, and as excellent (≥0.75), good (0.60–0.74), fair (0.40–0.59), or poor (<0.40) for ICC. The result of agreement analysis is shown in **Online Resource 4**.

**Online Resource 4. Inter- and Intra-Rater Agreement in Imaging Evaluation**

| **Item** | **Agreement Measure** | **Intra-rater Agreement(95%CI)** | **Inter-rater Agreement (95%CI)** |
| --- | --- | --- | --- |
| ASPECTS | ICC | 0.94(0.87-0.98) | 0.88(0.75-0.95) |
| Infarct size | Kappa | 0.89(0.54-0.99) | 0.83(0.49-0.99) |
| MBE | Kappa | 0.86(0.46-0.99) | 0.86(0.46-0.99) |

CI, confidence interval; ASPECTS, Alberta Stroke Program Early CT Score; MBE, malignant brain edema; ICC, intraclass correlation coefficient.

**Online Resource 5. Full Statistical Methods**

We assessed data normality by evaluating skewness and kurtosis, combining them into an overall statistic[10]. Continuous variables were presented as mean ± standard deviation (SD) for normally distributed data or median (interquartile range, IQR) otherwise. Categorical variables were reported as frequencies and percentages. Student’s t-test or Mann-Whitney U test was used for continuous variables, and Chi-square or Fisher’s exact test was applied for categorical data, depending on sample size. To assess the distribution of three-month mRS scores between groups, we additionally applied performed ordinal shift analysis.

We applied generalized estimating equation (GEE) to compare temporal profile of blood pressure level over the 24h recording period after stroke onset between MBE and non-MBE groups. We performed multivariable logistic regression model to analyze the independent associations between blood pressure parameters and MBE development, selecting confounders based on univariable analysis (*P*<0.1), clinical constraints, and prior research. To address collinearity, we applied stepwise backward elimination, sequentially removing variables with *P*>0.1 based on t-tests (for linear regression) or Wald tests (for logistic regression). Key clinically relevant variables (e.g., age) were retained regardless of statistical significance. Results were reported as odds ratio (OR) with 95% confidence interval (CI). Unless otherwise specified, the mean, maximum, minimum, and range of BP were included as continuous variables in the multivariable logistic regression analysis, with ORs indicating the increase in odds for every 1 mmHg increase in these variables.

We used restricted cubic splines (RCS) analysis[11] to examine the dose-response relationship between blood pressure parameters and MBE risk. A nonlinearity test was performed to determine the functional form of the association. Spline knots were placed at the 5^th^, 35^th^, 65^th^, and 95^th^ percentiles. The 25^th^ percentile or the inflection point of the RCS curve served as the reference, with an assigned OR of 1.

We predefined four subgroups based on mechanical thrombectomy status and reperfusion success: (1) patients who underwent thrombectomy, (2) patients who did not undergo thrombectomy, (3) patients who achieved successful reperfusion after thrombectomy, and (4) patients who either did not undergo thrombectomy or underwent thrombectomy but did not achieve successful reperfusion. Successful reperfusion was defined as a modified Thrombolysis in Cerebral Infarction (mTICI) score of 2b/3 post-thrombectomy[8]. Within each subgroup, we performed multivariable logistic regression to examine the association between blood pressure parameters and MBE risk, adjusting for relevant covariates.

All statistical tests were two-sided, with *P*≤0.05 considered statistically significant. Analyses were conducted using Stata 16.0 (Stata Corp, TX, USA) and SPSS 26.0 (IBM Corp, USA).

**
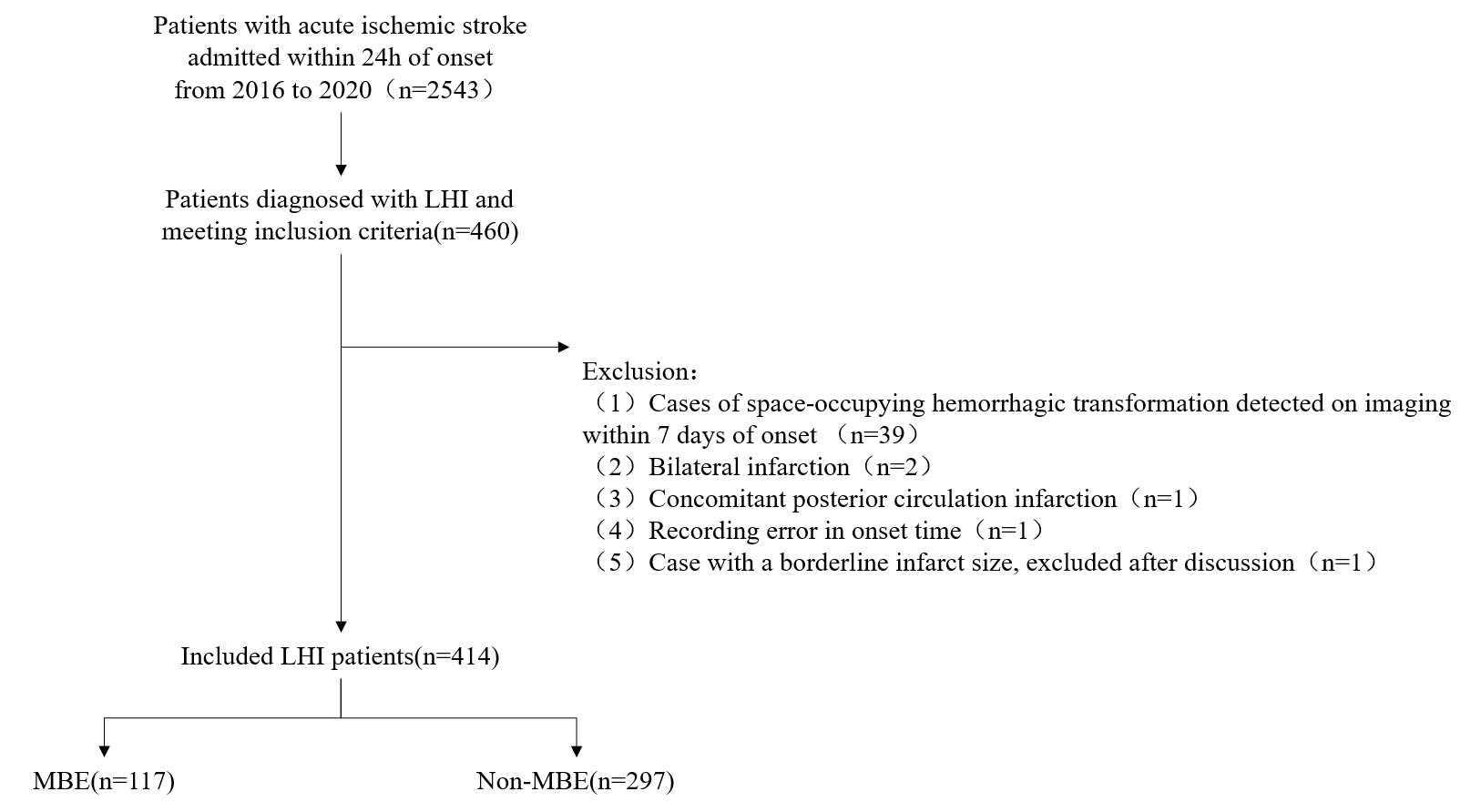
**

**Online Resource 6. Flowchart of Patient Inclusion**

LHI, large hemispheric infarction; MBE, malignant brain edema.

**Online Resource 7. Demographic Characters of Included Patients**

|  | **All(n=414)** | **MBE(n=117)** | **Non-MBE (n=297)** | ***P* value*** |
| --- | --- | --- | --- | --- |
| Male, n (%) | 207(50.00) | 46(39.32) | 161(54.21) | **0.006** |
| Age, year, mean±sd | 69±14 | 69±13 | 70±14 | 0.460 |
| Onset to admission, hour, median (IQR) | 3.12(2-5) | 3(1.88-4) | 3.23(2-5) | 0.060 |
| Onset to LHI diagnosis, hour, median (IQR) | 12.92  (4.82-31.32) | 6.68  (3.88-23.05) | 17.12  (5.87-35.05) | **<0.001** |
| Ischemic area, n (%) |  |  |  | **<0.001** |
| 1/3-1/2 MCA territory | 166(40.10) | 36(30.77) | 130(43.77) |  |
| 1/2-2/3 MCA territory | 132(31.88) | 28(23.93) | 104(35.02) |  |
| >2/3 MCA territory | 116(28.02) | 53(45.30) | 63(21.21) |  |
| Admission ASPECTS, median (IQR) | 6(4-8) | 5(3-8) | 6(4-8) | **0.007** |
| NIHSS, median (IQR) | 16(12-20) | 18(13-21) | 15(12-20) | **0.005** |
| Hypertension, n (%) | 206(49.76) | 63(53.85) | 143(48.15) | 0.296 |
| Diabetes, n (%) | 59(14.25) | 20(17.09) | 39(13.13) | 0.299 |
| Hyperlipidemia, n (%) | 15(3.62) | 6(5.13) | 9(3.03) | 0.304 |
| Atrial fibrillation, n (%) | 120(28.99) | 36(30.77) | 84(28.28) | 0.616 |
| Coronary heart disease, n (%) | 10(2.42) | 3(2.56) | 7(2.36) | 0.902 |
| TOAST classification, n (%) |  |  |  | 0.629 |
| Large-artery atherosclerosis | 161(38.89) | 45(38.46) | 116(39.06) |  |
| Small-vessel Occlusion | 0(0.0) | 0(0.0) | 0(0.0) |  |
| Cardioembolic | 191(46.14) | 58(49.57) | 133(44.78) |  |
| Other determined Etiology | 10(2.42) | 3(2.56) | 7(2.36) |  |
| Undetermined etiology | 52(12.56) | 11(9.40) | 41(13.80) |  |
| Thrombolysis, n (%) | 65(15.70) | 18(15.38) | 47(15.82) | 0.912 |
| Thrombectomy, n (%) | 132(31.88) | 46(39.32) | 86(28.96) | **0.042** |
| Successful reperfusion, n (%) | 103(24.88) | 32(27.35) | 71(72.65) | 0.465 |
| Dehydration therapy, n (%) | 357(86.23) | 112(95.73) | 245(82.50) | **<0.001** |
| Pneumonia, n (%) | 285(68.84) | 90(76.92) | 195(65.66) | **0.026** |
| Urinary tract infection, n (%) | 28(6.76) | 6(5.13) | 22(7.41) | 0.406 |
| Three-month mRS score, median (IQR) | 4(2-6) | 6(4-6) | 4(2-5) | **<0.001** |

*Student’s t-test or Mann-Whitney U test was used for continuous variables, and Chi-square or Fisher’s exact test was applied for categorical data, depending on sample size. To assess the distribution of three-month mRS scores between groups, we performed ordinal shift analysis.

SD, standard deviation；IQR, interquartile range; MCA，middle cerebral artery；ASPECTS，Alberta Stroke Programmed Early CT Score；NIHSS，National Institutes of Health Stroke Scale；TOAST，the Trial of Org 10172 in Acute Stroke Treatment; mRS, modified Rankin Scale.


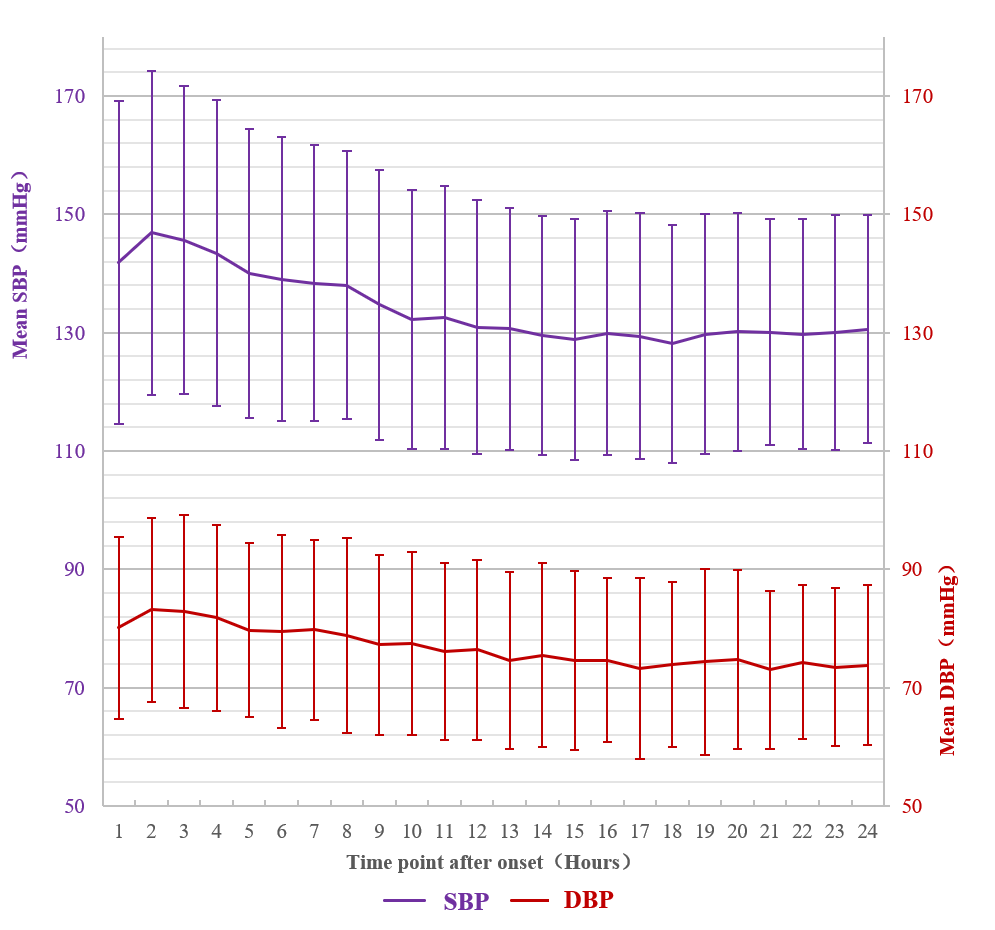


**Online Resource 8. Overall Trend of Hourly Blood Pressure Measurements during 24h after Large Hemispheric Infarction**

The upper and lower error bars represent the range of the mean ± one standard deviation. SBP, systolic blood pressure; DBP, diastolic blood pressure.

##### Online Resource 9. Comparison of SBP Parameters Between Patients with and without MBE after Large Hemispheric Infarction

|  | **MBE(n=117)** | **Non-MBE(n=297)** | ***P* value** |
| --- | --- | --- | --- |
| **24h post-stroke** |  |  |  |
| 24h blood pressure measurement count, pair, median (IQR) | 14(12-17) | 15(12-18) | 0.779 |
| First SBP, mmHg, median(IQR) | 141.00  (123.00-158.00) | 141.00  (125.00-159.00) | 0.521 |
| SBP mean, mmHg, median(IQR) | 132.50  (122.43-145.15) | 130.93  (121.73-143.00) | 0.288 |
| SBP mean>=140mmHg,n(%) | 48(41.03) | 87(29.29) | **0.022** |
| SBP mean>=160mmHg,n(%) | 8(6.84) | 13(4.38) | 0.304 |
| SBP mean>=180mmHg,n(%) | 0(0.0) | 2(0.67) | 0.374 |
| SBP max, mmHg, median(IQR) | 160.00  (144.00-178.00) | 157.00  (145.00-175.00) | 0.382 |
| SBP min, mmHg, median(IQR) | 108.00  (100.00-121.00) | 107.00  (98.00-118.00) | 0.419 |
| SBP max-min, mmHg, median(IQR) | 47.00(34.00-66.00) | 48.00(36.00-65.00) | 0.990 |
| **1-12h post-stroke** |  |  |  |
| 1-12h blood pressure measurement count, pair, median(IQR) | 7(5-9) | 7(5-8) | 0.972 |
| 1-12h SBP mean, mmHg, median(IQR) | 137.00  (125.39-150.88) | 133.66  (123.90-148.10) | 0.475 |
| 1-12h SBP mean>=140mmHg,n(%) | 52(44.44) | 129(43.43) | 0.852 |
| 1-12h SBP mean>=160mmHg,n(%) | 19(16.24) | 50(16.84) | 0.884 |
| 1-12hSBP mean>=180mmHg,n(%) | 6(5.13) | 26(8.75) | 0.214 |
| SBP max, mmHg, median(IQR) | 156.00  (141.50-178.00) | 155.50  (140.50-174.50) | 0.531 |
| SBP mean, mmHg, median(IQR) | 117.00  (104.00-131.00) | 115.00  (106.00-129.00) | 0.652 |
| SBP max-min, mmHg, median(IQR) | 34.50  (25.00-56.00) | 37.00  (26.00-52.00) | 0.762 |
| **13-24h post-stroke** |  |  |  |
| 13-24h blood pressure measurement count，pair, median(IQR) | 7(6-10) | 8(7-11) | **0.048** |
| 13-24h SBP mean, mmHg, mean(SD) | 131.45±17.30 | 128.95±16.20 | 0.173 |
| 13-24h SBP mean>=140mmHg,n(%) | 40(34.19) | 81(27.27) | 0.164 |
| 13-24h SBP mean>=160mmHg,n(%) | 13(11.11) | 12(4.04) | **0.007** |
| 13-24h SBP mean>=180mmHg,n(%) | 7(5.98) | 2(0.67) | **0.003** |
| SBP max, mmHg, mean±sd | 147.54±20.94 | 144.27±17.68 | 0.117 |
| SBP min, mmHg, median(IQR) | 112.50  (102.00-128.00) | 112.00  (102.00-124.00) | 0.337 |
| SBP max-min, mmHg, median(IQR) | 30.50  (21.00-39.00) | 29.00  (21.00-38.00) | 0.708 |

SBP, systolic blood pressure; LHI, large hemispheric infarction; MBE, malignant brain edema; SD, standard deviation；IQR, Interquartile range; CV, coefficient of variation.

##### Online Resource 10. Multivariate Logistic Analysis of SBP Parameters and MBE Risk after Large Hemispheric Infarction

|  | **Adjusted OR*** | **95%CI** | ***P* value** |
| --- | --- | --- | --- |
| **24h post-stroke** |  |  |  |
| First SBP | 1.00 | 0.99-1.01 | 0.644 |
| Mean SBP | 1.02 | 1.00-1.04 | **0.031** |
| Mean SBP>=140mmHg | 2.81 | 1.63-4.84 | **<0.001** |
| Mean SBP>=160mmHg | 1.80 | 0.64-5.02 | 0.262 |
| Mean SBP>=180mmHg | Unable to calculate | | |
| SBP max | 1.00 | 0.99-1.01 | 0.625 |
| SBP min | 1.02 | 1.00-1.03 | **0.022** |
| SBP max-min | 0.99 | 0.98-1.00 | 0.253 |
| **1-12h post-stroke** |  |  |  |
| 1-12h SBP mean | 1.01 | 0.99-1.02 | 0.270 |
| 1-12h SBP mean>=140mmHg | 1.25 | 0.75-2.08 | 0.394 |
| 1-12h SBP mean>=160mmHg | 1.61 | 0.81-3.20 | 0.172 |
| 1-12h SBP mean>=180mmHg | 1.32 | 0.43-4.03 | 0.625 |
| SBP max | 1.00 | 0.99-1.01 | 0.716 |
| SBP min | 1.01 | 1.00-1.02 | 0.131 |
| SBP max-min | 0.99 | 0.98-1.01 | 0.389 |
| **13-24h post-stroke** |  |  |  |
| 13-24h SBP mean | 1.02 | 1.00-1.04 | **0.013** |
| 13-24h SBP mean>=140mmHg | 1.82 | 1.06-3.13 | **0.030** |
| 13-24h SBP mean>=160mmHg | 2.98 | 1.21-7.31 | **0.017** |
| 13-24h SBP mean>=180mmHg | 9.29 | 1.68-51.49 | **0.011** |
| SBP max | 1.02 | 1.00-1.03 | **0.009** |
| SBP mean | 1.01 | 1.00-1.03 | **0.044** |
| SBP max-mean | 1.01 | 0.99-1.02 | 0.468 |

* We performed binary stepwise backward logistic regression analysis, incorporating baseline factors with *P* < 0.1 from the univariate analysis and factors with clinical constraints. Variables not significantly contributing to the model were systematically removed. We manually retained variables with strong clinical importance, such as age, despite their exclusion based on statistical criteria. We identified factors most closely associated with MBE, including age, sex, onset-to-admission interval, infarct size, NIHSS score, thrombectomy, and dehydration therapy for adjusting. SBP, systolic blood pressure; MBE, malignant brain edema; SD, standard deviation; CV, coefficient of variation；OR, odds ratio；CI, confidence interval. NIHSS, National Institutes of Health Stroke Scale

##### Online Resource 11 . Subgroup Analysis for Associations between SBP Parameters and MBE after Large Hemispheric Infarction

| **Subgroups** | **Adjusted OR** | **95%CI** | ***P* value** |
| --- | --- | --- | --- |
| **Thrombectomy** |  |  |  |
| 24h mean SBP>=140mmHg | 6.12^*^ | 2.02-18.55 | **0.001** |
| 13-24h mean SBP | 1.04^*^ | 1.00-1.07 | **0.039** |
| 13-24h SBP max | 1.04^*^ | 1.01-1.07 | **0.017** |
| **Non-thrombectomy** |  |  |  |
| 24h mean SBP>=140mmHg | 2.04^#^ | 1.08-3.85 | **0.027** |
| 13-24h mean SBP | 1.02^#^ | 1.00-1.04 | **0.045** |
| 13-24h SBP min | 1.02^#^ | 1.00-1.04 | **0.039** |
| **Successful reperfusion** |  |  |  |
| 24h mean SBP>=140mmHg | 5.40^§^ | 1.60-18.24 | **0.007** |
| **Unsuccessful reperfusion+ Non-thrombectomy** |  |  |  |
| 24h mean SBP>=140mmHg | 1.81^#^ | 1.01-3.26 | **0.047** |
| 24h mean SBP min | 1.02^#^ | 1.00-1.04 | **0.043** |
| 13-24h SBP mean | 1.02^#^ | 1.00-1.04 | **0.034** |
| 13-24h SBP min | 1.02^#^ | 1.00-1.04 | **0.014** |

^*^Adjusted by age, sex, infarct size, NIHSS

^#^Adjusted by age, sex, infarct size, NIHSS, onset-to-admission interval, pneumonia

^§^Adjusted by age, infarct size

SBP, systolic blood pressure; MBE, malignant brain edema; OR, odds ratio; CI, confidence interval; NIHSS, National Institutes of Health Stroke Scale

**
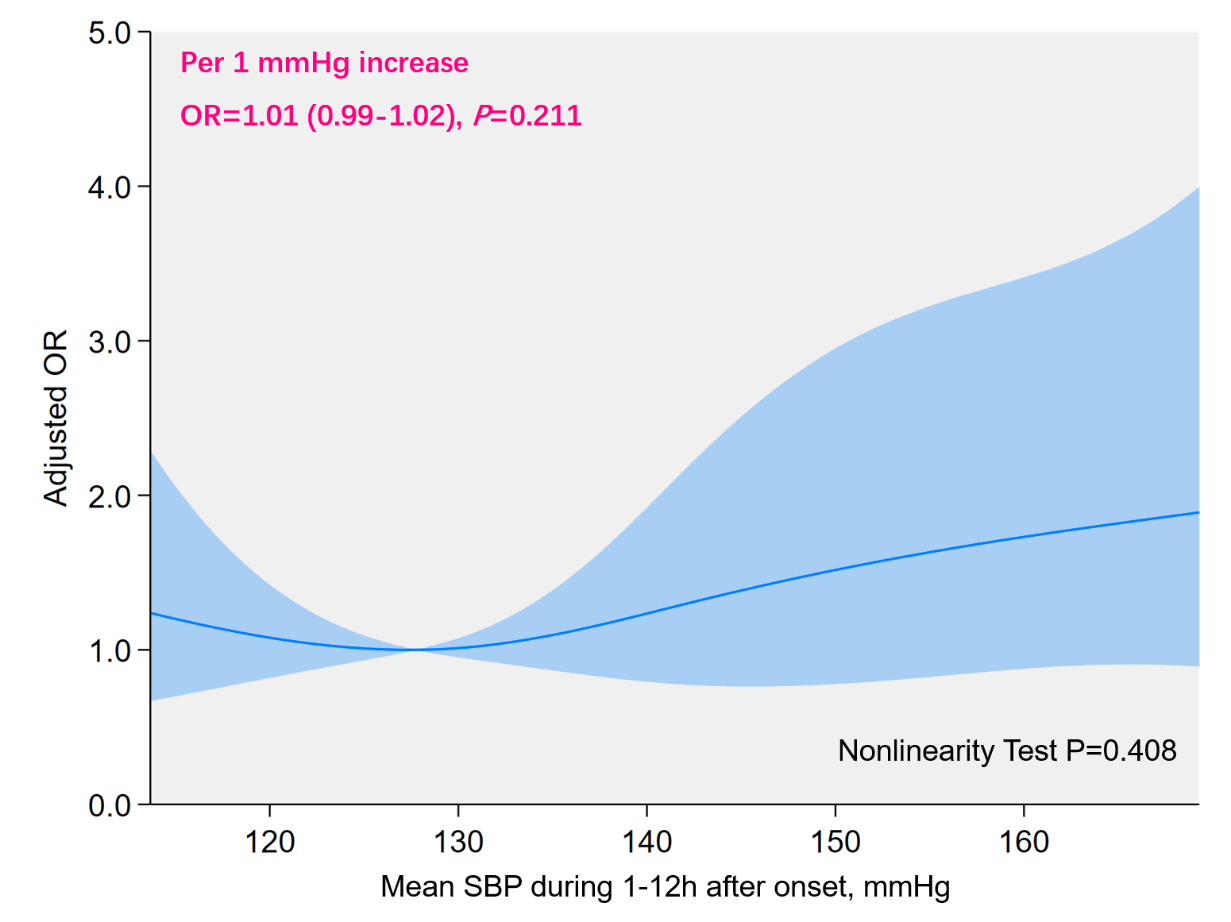
**

**Online Resource 12. Association between SBP and MBE Risk in Patients with Large Hemispheric Infarction during 1-12h after Onset**

The vertical axis represented adjusted OR, adjusted by age, sex, onset-to-admission interval, infarct size, NIHSS score, thrombectomy, and dehydration therapy. Solid blue line indicated OR values, while light blue shaded area represented the 95% confidence interval. The reference point was at an x-axis value of 120 mmHg, with spline knots positioned at the 5th, 35th, 65th, and 95th percentiles. SBP, systolic blood pressure; OR, odds ratio; NIHSS, National Institutes of Health Stroke Scale.


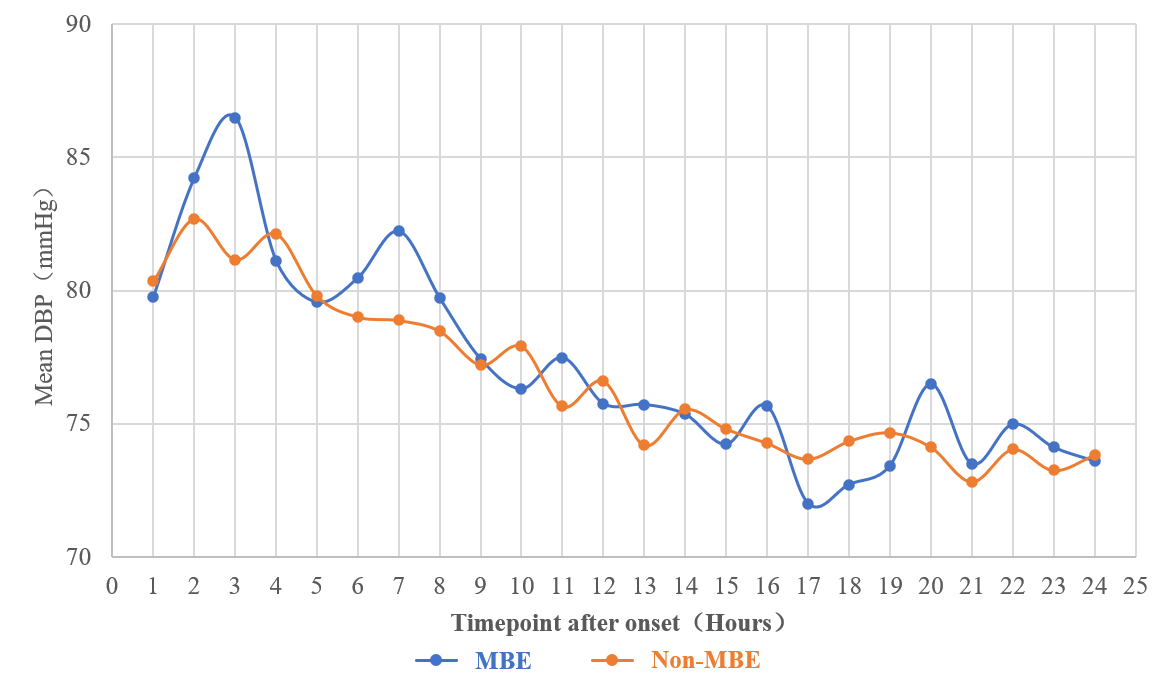
**Online Resource 13. Overall DBP Pattern during 24h after Large Hemispheric Infarction**

DBP, diastolic blood pressure; MBE, malignant brain edema

##### Online Resource 14. Comparison of DBP Parameters Between Patients with and without MBE after Large Hemispheric Infarction

|  | MBE  (n=117) | Non-MBE (n=297) | *P* value |
| --- | --- | --- | --- |
| **24h post-stroke** |  |  |  |
| 24h blood pressure measurement count, pair, median (IQR) | 14(12-17) | 15(12-18) | 0.779 |
| First DBP, mmHg, median (IQR) | 83.00(72.00-99.00) | 83.00(72.00-94.00) | 0.600 |
| Mean DBP, mmHg, median (IQR) | 77.25 (69.81-82.94) | 75.35 (69.70-81.93) | 0.321 |
| DBP mean>=90mmHg, n (%) | 12(10.26) | 32(10.77) | 0.878 |
| DBP mean>=100mmHg, n (%) | 5(4.27) | 7(2.36) | 0.295 |
| DBP mean>=110mmHg, n (%) | 0(0.0) | 2(0.67) | 0.374 |
| DBP max, mmHg, median (IQR) | 98.00(89.00-108.00) | 95.00(87.00-103.00) | 0.113 |
| DBP min, mmHg, median (IQR) | 60.00(53.00-67.00) | 60.00(53.00-66.00) | 0.888 |
| DBP max-min, mmHg, median (IQR) | 37.00(27.00-46.00) | 35.00(27.00-43.00) | 0.279 |
| **1-12h post-stroke** |  |  |  |
| 1-12h blood pressure measurement count, pair, median (IQR) | 7(5-9) | 7(5-8) | 0.972 |
| 1-12h DBP mean, mmHg, median (IQR) | 79.28 (72.58-87.38) | 77.59 (72.35-85.70) | 0.235 |
| 1-12h DBP mean>=90mmHg, n (%) | 28(23.93) | 60(20.20) | 0.404 |
| 1-12h DBP mean>=100mmHg, n (%) | 14(11.97) | 36(12.12) | 0.965 |
| 1-12h DBP mean>=110mmHg, n (%) | 10(8.55) | 23(7.74) | 0.786 |
| DBP max, mmHg, median (IQR) | 94.50(85.00-107.00) | 93.00(85.00-102.00) | 0.258 |
| DBP min, mmHg, median (IQR) | 66.00(59.00-72.50) | 65.00(58.50-74.00) | 0.885 |
| DBP max-min, mmHg, median (IQR) | 27.50(19.00-38.50) | 27.00(19.00-36.00) | 0.653 |
| **13-24h post-stroke** |  |  |  |
| 13-24h blood pressure measurement count, pair, median (IQR) | 7(6-10) | 8(7-11) | **0.048** |
| 13-24h DBP mean, mmHg, median (IQR) | 73.81 (66.00-81.00) | 73.13 (67.00-81.13) | 0.980 |
| 13-24h DBP mean>=90mmHg, n (%) | 15(12.82) | 24(8.08) | 0.137 |
| 13-24h DBP mean>=100mmHg, n (%) | 11(9.4) | 8(2.69) | **0.003** |
| 13-24h DBP mean >=110mmHg, n (%) | 7(5.98) | 4(1.35) | **0.008** |
| DBP max, mmHg, median (IQR) | 88.00(76.00-98.00) | 85.00(78.00-94.00) | 0.261 |
| DBP min, mmHg, median (IQR) | 61.00(55.00-70.00) | 62.00(55.00-69.00) | 0.970 |
| DBP max-min, mmHg, median (IQR) | 23.00(15.00-36.00) | 23.00(17.00-31.00) | 0.543 |

DBP, diastolic blood pressure; MBE, malignant brain edema; SD, standard deviation；IQR, interquartile range; CV, coefficient of variation.

##### Online Resource 15. Multivariate Logistic Analysis of DBP Parameters and MBE Risk after Large Hemispheric Infarction

|  | Adjusted OR* | 95%CI | *P* value |
| --- | --- | --- | --- |
| **24h post-stroke** |  |  |  |
| First DBP | 1.00 | 0.99-1.02 | 0.487 |
| Mean DBP | 1.02 | 1.00-1.05 | **0.049** |
| Mean DBP>=140mmHg | 1.39 | 0.64-3.00 | 0.402 |
| Mean DBP>=160mmHg | 3.25 | 0.91-11.66 | 0.070 |
| Mean DBP>=180mmHg | Unable to calculate | | |
| DBP max | 1.01 | 0.99-1.02 | 0.337 |
| DBP min | 1.02 | 1.00-1.04 | 0.094 |
| DBP max-min | 1.00 | 0.98-1.01 | 0.881 |
| **1-12h post-stroke** |  |  |  |
| 1-12h DBP mean | 1.02 | 1.00-1.04 | **0.039** |
| 1-12h DBP mean>=140mmHg | 2.12 | 1.14-3.95 | **0.017** |
| 1-12h DBP mean>=160mmHg | 2.22 | 0.98-5.01 | 0.056 |
| 1-12h DBP mean>=180mmHg | 3.18 | 1.13-8.89 | **0.028** |
| DBP max | 1.01 | 0.99-1.02 | 0.286 |
| DBP min | 1.02 | .00-1.04 | 0.095 |
| DBP max-min | 1.00 | 0.98-1.02 | 0.832 |
| **13-24h post-stroke** |  |  |  |
| 13-24h DBP mean | 1.01 | 0.99-1.04 | 0.216 |
| 13-24h DBP mean>=140mmHg | 2.15 | 0.99-4.66 | 0.053 |
| 13-24h DBP mean>=160mmHg | 5.08 | 1.77-14.55 | **0.002** |
| 13-24h DBP mean>=180mmHg | 5.29 | 1.32-21.22 | **0.019** |
| DBP max | 1.01 | 1.00-1.03 | 0.146 |
| DBP mean | 1.01 | 0.99-1.04 | 0.253 |
| DBP max-mean | 1.01 | 0.99-1.02 | 0.553 |

*We performed binary stepwise backward logistic regression analysis, incorporating baseline factors with *P* < 0.1 from the univariate analysis and factors with clinical constraints. Variables not significantly contributing to the model were systematically removed. We manually retained variables with strong clinical importance, such as age, despite their exclusion based on statistical criteria. We identified factors most closely associated with MBE, including age, sex, onset-to-admission interval, infarct size, NIHSS score, thrombectomy, and dehydration therapy for adjusting. SD, standard deviation; CV, coefficient of variation; OR, odds ratio; CI, confidence interval; NIHSS, National Institutes of Health Stroke Scale

##### Online Resource 16. Subgroup Analysis for Associations between DBP Parameters and MBE after Large Hemispheric Infarction

| **Subgroups** | **Adjusted OR** | **95%CI** | ***P* value** |
| --- | --- | --- | --- |
| **Thrombectomy** |  |  |  |
| No significant result^*^ | | | |
| **Non-thrombectomy** |  |  |  |
| 1-12h mean SBP | 1.03^#^ | 1.00-1.05 | **0.034** |
| 1-12h SBP max | 1.02^#^ | 1.00-1.04 | **0.030** |
| **Successful reperfusion** |  |  |  |
| No significant result^§^ | | | |
| **Unsuccessful reperfusion+ Non-thrombectomy** |  |  |  |
| 1-12h mean SBP | 1.03^#^ | 1.00-1.05 | **0.038** |
| 1-12h SBP max | 1.02^#^ | 1.00-1.04 | **0.033** |

^*^Adjusted by age, sex, infarct size, NIHSS

^#^Adjusted by age, sex, infarct size, NIHSS, onset-to-admission interval, dehydration therapy

^§^Adjusted by age, infarct size

DBP, diastolic blood pressure; MBE, malignant brain edema; OR, odds ratio; CI, confidence interval; NIHSS, National Institutes of Health Stroke Scale.


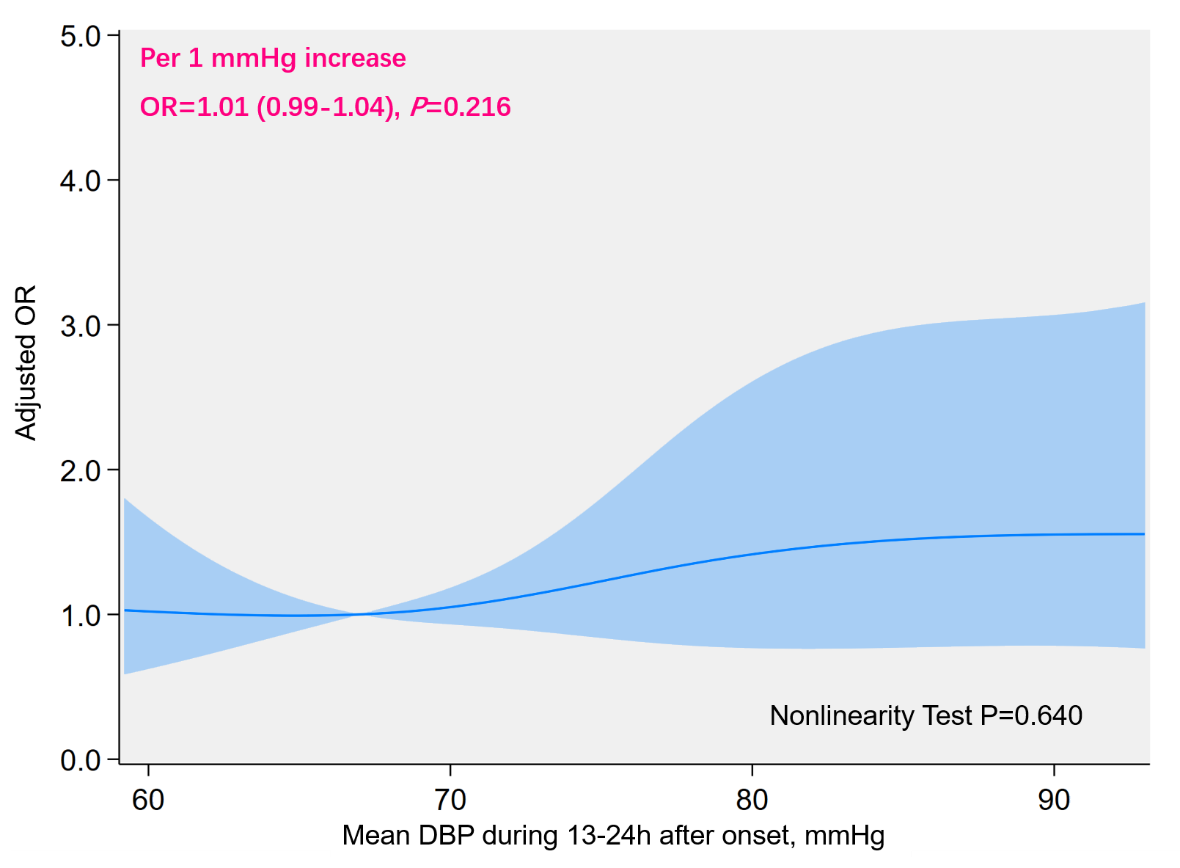


**Online Resource 17. Association between DBP and MBE Risk in Patients with Large Hemispheric Infarction during 13-24h after Onset**

The vertical axis represented adjusted OR, adjusted by age, sex, onset-to-admission interval, infarct size, NIHSS score, thrombectomy, and dehydration therapy. Solid blue line indicated OR values, while light blue shaded area represented the 95% confidence interval. The reference point was at an x-axis value of 70 mmHg, with spline knots positioned at the 5th, 35th, 65th, and 95th percentiles. DBP, diastolic blood pressure; OR, odds ratio; NIHSS, National Institutes of Health Stroke Scale.

1. Williams, B., et al., 2018 ESC/ESH Guidelines for the management of arterial hypertension: The Task Force for the management of arterial hypertension of the European Society of Cardiology and the European Society of Hypertension: The Task Force for the management of arterial hypertension of the European Society of Cardiology and the European Society of Hypertension. J Hypertens, 2018. **36**(10): p. 1953-2041.<https://doi.org/10.1097/hjh.0000000000001940>

2. 2. Diagnosis and Classification of Diabetes: Standards of Care in Diabetes-2024. Diabetes Care, 2024. **47**(Suppl 1): p. S20-s42.<https://doi.org/10.2337/dc24-S002>

3. Kleindorfer, D.O., et al., 2021 Guideline for the Prevention of Stroke in Patients With Stroke and Transient Ischemic Attack: A Guideline From the American Heart Association/American Stroke Association. Stroke, 2021. **52**(7): p. e364-e467.<https://doi.org/10.1161/STR.0000000000000375>

4. Van Gelder, I.C., et al., 2024 ESC Guidelines for the management of atrial fibrillation developed in collaboration with the European Association for Cardio-Thoracic Surgery (EACTS). Eur Heart J, 2024. **45**(36): p. 3314-3414.<https://doi.org/10.1093/eurheartj/ehae176>

5. Virani, S.S., et al., 2023 AHA/ACC/ACCP/ASPC/NLA/PCNA Guideline for the Management of Patients With Chronic Coronary Disease: A Report of the American Heart Association/American College of Cardiology Joint Committee on Clinical Practice Guidelines. Circulation, 2023. **148**(9): p. e9-e119.<https://doi.org/10.1161/cir.0000000000001168>

6. Lyden, P., et al., Improved reliability of the NIH Stroke Scale using video training. NINDS TPA Stroke Study Group. Stroke, 1994. **25**(11): p. 2220-6.<https://doi.org/10.1161/01.str.25.11.2220>

7. Pexman, J.H., et al., Use of the Alberta Stroke Program Early CT Score (ASPECTS) for assessing CT scans in patients with acute stroke. AJNR Am J Neuroradiol, 2001. **22**(8): p. 1534-42

8. Powers, W.J., et al., Guidelines for the Early Management of Patients With Acute Ischemic Stroke: 2019 Update to the 2018 Guidelines for the Early Management of Acute Ischemic Stroke: A Guideline for Healthcare Professionals From the American Heart Association/American Stroke Association. Stroke, 2019. **50**(12): p. e344-e418.<https://doi.org/10.1161/str.0000000000000211>

9. Adams, H.P., Jr., et al., Classification of subtype of acute ischemic stroke. Definitions for use in a multicenter clinical trial. TOAST. Trial of Org 10172 in Acute Stroke Treatment. Stroke, 1993. **24**(1): p. 35-41.<https://doi.org/10.1161/01.str.24.1.35>

10. D'agostino, R.B., A. Belanger, and R.B. D'Agostino Jr, A suggestion for using powerful and informative tests of normality. The American Statistician, 1990. **44**(4): p. 316-321

11. Abell, J.G., et al., Association between systolic blood pressure and dementia in the Whitehall II cohort study: role of age, duration, and threshold used to define hypertension. Eur Heart J, 2018. **39**(33): p. 3119-3125.<https://doi.org/10.1093/eurheartj/ehy288>
